# Avian malaria parasites in the last supper: identifying encounters between parasites and the invasive Asian mosquito tiger and native mosquito species in Italy

**DOI:** 10.1186/s12936-015-0571-0

**Published:** 2015-01-28

**Authors:** Josué Martínez-de la Puente, Joaquín Muñoz, Gioia Capelli, Fabrizio Montarsi, Ramón Soriguer, Daniele Arnoldi, Annapaola Rizzoli, Jordi Figuerola

**Affiliations:** Departamento de Ecología de Humedales, Estación Biológica de Doñana (EBD-CSIC), C/Américo Vespucio s/n, Seville, E-41092 Spain; Istituto Zooprofilattico Sperimentale delle Venezie, Viale dell’Università, 10, 35020 Legnaro, Italy; Departamento de Etología y Conservación de la Biodiversidad, Estación Biológica de Doñana (EBD-CSIC), C/Américo Vespucio s/n, Seville, E-41092 Spain; Fondazione Edmund Mach, Centro Ricerca e Innovazione, 38010 San Michele all’Adige, TN Italy

**Keywords:** *Aedes albopictus*, Avian diseases, *Culex pipiens*, *Haemoproteus*, Mosquitoes, *Plasmodium*, Vector, West Nile virus

## Abstract

**Background:**

The invasive Asian tiger mosquito *Aedes albopictus* has dramatically expanded its distribution range, being catalogued as one of the world’s 100 worst invasive alien species. As vectors of pathogens, *Ae. albopictus* may create novel epidemiological scenarios in the invaded areas.

**Methods:**

Here, the frequency of encounters of *Ae. albopictus* with the avian malaria parasite *Plasmodium* and the related *Haemoproteus* was studied in an area with established populations in northeastern Italy and compared with those from four native mosquito species, *Anopheles maculipennis s.l., Culex hortensis*, *Culex pipiens*, and *Ochlerotatus caspius*. The abdomens of mosquitoes with a recent blood meal were used to identify both the blood meal source and the parasites harboured.

**Results:**

*Aedes albopictus* had a clear antropophilic behaviour while *An. maculipennis* and *Oc. caspius* fed mainly on non-human mammals. Birds were the most common hosts of *Cx. pipiens* and reptiles of *Cx. hortensis*. Parasites were isolated from three mosquito species, with *Cx. pipiens* (30%) showing the highest parasite prevalence followed by *Cx. hortensis* (9%) and *Ae. albopictus* (5%).

**Conclusions:**

These results are the first identifying the avian malaria parasites harboured by mosquitoes in Italy and represent the first evidence supporting that, although *Ae. albopictus* could be involved in the transmission of avian malaria parasites, the risk of avian malaria parasite spread by this invasive mosquito in Europe would be minimal.

## Background

Establishment of exotic mosquitoes to new areas may create novel epidemiological scenarios with potential dramatic consequences for wildlife and human health [1]. The invasive Asian tiger mosquito *Aedes albopictus*, indigenous to Southeast Asia, islands of the Western Pacific and Indian Ocean, has expanded its distribution range to Africa, Europe and the Americas [2,3]. In Europe, this species was first recorded in 1979 in Albania [4] and subsequently in Italy, France and other countries of the Mediterranean region and northern Europe [1,3]. *Aedes albopictus* is vector of a diversity of pathogens including flaviviruses (e.g., West Nile virus, Dengue virus), alphaviruses (e.g., Chikungunya virus), and other viruses and filarial worms [5,6]. In Europe, *Ae. albopictus* has been incriminated in the transmission of both introduced (Chikungunya and Dengue viruses) and endemic (*Dirofilaria* nematodes) pathogens [5,7].

Avian malaria parasites of the genus *Plasmodium,* and the relative haemosporidian *Haemoproteus*, produce pathogenic effects on both vertebrate and invertebrate hosts [8]. *Plasmodium* parasites require the intervention of a mosquito vector to be transmitted from an infected bird to another individual. *Haemoproteus* parasites have a similar life cycle, requiring a biting midge *Culicoides* or louse flies instead of mosquitoes to be transmitted between birds [8]. During a bite event a mosquito feeding on an infected bird is able to acquire the parasites contained in the blood. A number of mosquito species belonging to different genera such as *Aedes*, *Anopheles* and *Culex,* have been reported as potential vectors of avian malaria parasites [9]. Although different factors may influence the subsequent development of parasites in the mosquito after blood ingestion [10], pathogen isolation of recent blood meals may provide valuable information on parasite-mosquito encounters and potential parasite transmission [11,12]. In this respect, the blood ingested by potential vectors can be used as a source of host DNA to identify both the feeding sources of mosquitoes [13] and the blood parasites reaching these potential vectors [12].

In spite of their importance on parasite transmission, only a handful of studies have identified the blood parasites interacting with wild mosquito populations in Europe [14-18], and, no previous study has tested for the presence of avian malaria parasites in invasive populations of the tiger mosquito *Ae. albopictus*. Here, two molecular approaches were used to identify both host and avian malaria parasites from blood contained in the mosquito’s abdomen following protocols described by Alcaide *et al*. [13] and Hellgren *et al*. [19], respectively. Samples from five different mosquito species collected in northeastern Italy were included in this study: the invasive *Ae. albopictus*, and the native *Anopheles maculipennis s.l., Culex hortensis, Culex pipiens*, and *Ochlerotatus caspius*.

## Methods

### Mosquito sampling and morphological identification

Mosquitoes were collected from May to October 2012 using BG-sentinel traps baited with BG-lure and dry ice. Twenty traps in Veneto and ten in Trentino provinces operated once a week or two weeks for 24 hr, respectively. In Trentino mosquitoes were also collected using a motor-powered aspirator. Mosquitoes were morphologically identified following the keys of the Italian Culicidae adults [20] and preserved frozen (−20 or −80°C) until examination.

### Blood meal source and parasite identification

DNA from the abdomen of blood-fed mosquitoes was individually isolated using the DNeasy Blood and Tissue® kit (QIAGEN, Hilden, Germany) following company specifications. This DNA extraction approach resulted in a higher efficacy of host identification than other protocols such as Hotshot [21]. Vertebrate blood meal origin was identified using a nested-PCR approach [13] to amplify a 758-base pairs fragment of the mitochondrial cytochrome oxidase 1 (COI) gene. Both negative controls for PCR reactions (at least one per plate) and DNA extraction were included in the analysis. DNA was also used to identify the presence of *Plasmodium* and *Haemoproteus* parasites based on the amplification of a fragment of the mitochondrial Cytochrome b gene [19].

Positive amplifications were sequenced using the Big Dye 1.1 technology (Applied Biosystems). Labelled DNA fragments of positive PCR products were resolved with an ABI 3130xl automated sequencer (Applied Biosystems) using the same forward and reverse primers used in the nested-PCR amplification for the case of blood parasite identifications. For blood meal identifications, amplicons were sequenced in one direction using the primer BCRV2, except for the case of *Ae. albopictus* mosquitoes that were sequenced using the primer BCVINT-RV (see [22]). Sequences were edited using the software Sequencher™ v 4.9 (Gene Codes Corp., © 1991–2009, Ann Arbor, MI 48108, USA). Blood meal sequences were assigned to particular vertebrate species when agreement was ≥98% to sequences of known species in GenBank DNA sequence database (National Center for Biotechnology Information Blast) or the Barcode of Life Data Systems (BOLD). Parasite lineages were identified by comparison with sequences deposited in GenBank database. Statistical significance of differences in parasite prevalence was tested with statistical software JMP (version 9.0.1).

## Results

**Figure 1:**
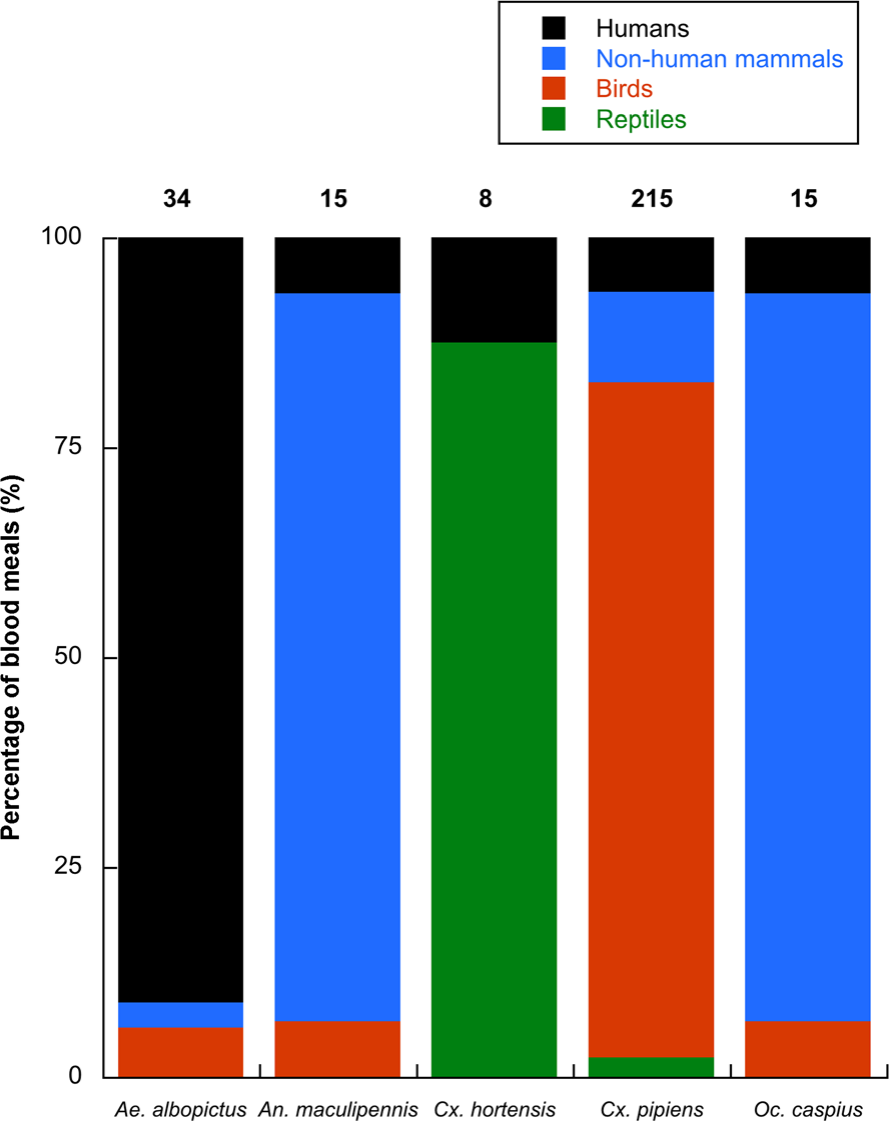
**Source of mosquito blood meals of the five mosquito species analysed.** Numbers above bars indicate the number of blood meals per mosquito species identified. Mixed blood meals from more than one host species were excluded.

Overall, 348 blood-fed mosquitoes belonging to five different species were included in this study. The most extensively species sampled was *Cx. pipiens* (n = 264), followed by *Ae. albopictus* (n = 41), *An. maculipennis* (n = 16), *Oc. caspius* (n = 16) and *Cx. hortensis* (n = 11). The blood meal source of 290 (83.3%) of them was successfully identified, compromising, at least, 36 vertebrate species including 11 mammals, 23 birds and two reptiles (Table 1). Blackbirds *Turdus merula* was the most common host species of mosquitoes compromising 73 blood meals. Clear differences in mosquito feeding sources were found among mosquito species (Figure 1). Three mosquitoes showed evidence of mixed blood meals tentatively identified: *H. sapiens* + *Columba livia*, *H. sapiens* + *Gallus gallus* and *H. sapiens* + *Felis silvestris*/*catus*.

**Table 1:** **Blood meal source of mosquitoes in Italy**

**Mosquito species**	**Mammal**	**Bird**	**Reptile**
*Ae. albopictus*	*Homo sapiens* (31)	*Passer montanus* (1)	
	*Erinaceus europaeus* (1)	*Turdus merula* (1)	
*An. maculipennis*	*Canis lupus familiares* (3)	*Gallus gallus* (1)	
	*Equus asinus* (2)		
	*Equus caballus* (2)		
	*Lepus europaeus* (2)		
	*Bos taurus* (1)		
	*Capra hircus* (1)		
	*Felis silvestris*/*catus* (1)		
	*Homo sapiens* (1)		
	*Vulpes vulpes* (1)		
*Cx. hortensis*	*Homo sapiens* (1)		*Podarcis muralis* (7)
*Cx. pipiens*	*Homo sapiens* (14)	*Turdus merula* (72)	*Podarcis muralis* (4)
	*Felis silvestris*/*catus* (9)	*Passer domesticus* (26)	*Lacerta spp*. (1)
	*Canis lupus familiaris* (5)	*Gallus gallus* (21)	
	*Equus caballus* (3)	*Streptopelia decaocto* (16)	
	*Sus scrofa* (3)	*Columba livia* (5)	
	*Bos taurus* (2)	*Passer montanus* (5)	
	*Erinaceus europaeus* (1)	*Athene noctua* (4)	
		*Meleagris gallopavo* (3)	
		*Columba palumbus* (3)	
		*Pica pica* (3)	
		*Anas platyrhychos* (2)	
		*Sturnus vulgaris* (2)	
		*Accipiter nisus* (1)	
		*Cairina moschata* (1)	
		*Carduelis carduelis* (1)	
		*Gallinula chloropus* (1)	
		*Jynx torquilla* (1)	
		*Numida meleagris* (1)	
		*Nycticorax nycticorax* (1)	
		*Oriolus oriolus* (1)	
		*Parus major* (1)	
		*Phasianus colchinus* (1)	
		*Sylvia atricapilla* (1)	
*Oc. caspius*	*Felis silvestris*/*catus* (6)	*Gallus gallus* (1)	
	*Equus asinus* (3)		
	*Equus caballus* (2)		
	*Bos taurus* (1)		
	*Canis lupus familiaris* (1)		
	*Homo sapiens* (1)		

Mixed blood meals from more than one host species were excluded.

Parasite infection status differed among mosquito species (χ^2^ = 35.78, d.f. = 4, p < 0.001) with *Cx. pipiens* showing higher prevalence of infection (30%, 80 infected out of 264 tested) than *Cx. hortensis* (9%, 1/11) and *Ae. albopictus* (5%, 2/41) (Table 2). Blood parasites were not found in *An. maculipennis* nor *Oc. caspius*. With the exception of two mosquitoes with blood meals from reptiles, the rest of the parasites detected corresponded to mosquitoes containing an avian-derived blood meal (Table 3). Seven out of 58 mosquito abdomens with too degraded blood to allow blood meal origin identification showed parasite positive amplifications. Sequences with double peaks in the chromatogram were obtained from four mosquitoes, probably reflecting the presence of more than one parasite lineages. Six *Plasmodium* and four *Haemoproteus* lineages were isolated from mosquitoes (Tables 2,3). The lineages identified were the *Plasmodium* lineages: SGS1 (also called Rinshi-1, belonging to *Plasmodium relictum*, n = 9), LINN1 (also called pSPHUjJ, n = 10), SYAT05 (also called Rinshi-11, belonging to *Plasmodium vaughani*, n = 47), Delurb4 (n = 2), GRW11 (also called Rinshi-7, belonging to *Plasmodium relictum*, n = 1) and Aftru5 (n = 1). The *Haemoproteus* lineages isolated were TURDUS2 (also called Bolin1, belonging to *Haemoproteus minutus*, n = 3), Padom3 (n = 2), hItCxpip01 (n = 2) and hCIRCUM05 (n = 1). The *Haemoproteus* lineage hItCxpip01 [GenBank: KP120693], described here for the first time, was isolated from two mosquitoes with blood from magpies *Pica pica*.

**Table 2:** **Blood parasite lineages isolated from mosquito blood meals**

	***Plasmodium***						***Haemoproteus***			
	**AFTRU5**	**LINN1**	**Delurb4**	**SGS1**	**GRW11**	**SYAT05**	**TURDUS2**	**hCIRCUM05**	**hItCxpip01**	**Padom3**
***Cx. pipiens***	**1**	**9**	**2**	**9**	**1**	**46**	**4**	**1**	**2**	**2**
***Cx. hortensis***		**1**								
***Ae. albopictus***						**1**

**Table 3:** **Blood meal source of mosquitoes harbouring identified blood parasite lineages**

**Parasite genus**	**Lineage**	**Mosquito species**	**Hosts**	**Times isolated**	**Known distribution**
*Plasmodium*	AFTRU5	*Cx. pipiens*	*Turdus merula*	1	Africa, Asia, Europe*
	Delurb4	*Cx. pipiens*	*Numida meleagris*	1	Asia, Europe
			*Passer domesticus*	1	
	GRW11	*Cx. pipiens*	*Passer domesticus*	1	Africa, Asia, Europe
	LINN1	*Cx. pipiens*	*Turdus merula*	6	Asia, Europe, Oceanía**
			*Passer montanus*	1	
			*Athene noctua*	1	
		*Cx. hortensis*	*Podarcis muralis*	1	
	SGS1	*Cx. pipiens*	*Passer domesticus*	4	Africa, America, Asia, Europe, Oceania
			*Passer montanus*	1	
			*Turdus merula*	1	
			*Gallus gallus*	1	
	SYAT05	*Cx. pipiens*	*Turdus merula*	41	Africa, America, Asia, Europe, Oceanía
			*Meleagris gallopavo*	1	
			*Passer domesticus*	2	
			*Podarcis muralis*	1	
		*Ae. albopictus*	*Turdus merula*	1	
*Haemoproteus*	TURDUS2	*Cx. pipiens*	*Turdus merula*	3	America, Asia, Europe
	hCircum05	*Cx. pipiens*	*Pica pica*	1	Europe
	hItCxpip01	*Cx. pipiens*	*Pica pica*	2	Europe
	Padom3	*Cx. pipiens*	*Passer montanus*	1	Europe

Previous known distribution of parasites according to Malavi database is recorded [36]. *Aftru5 was detected in birds in captivity in Oceania. **LINN1 was also isolated from whole unengorged mosquitoes in America [25].

## Discussion

Avian *Plasmodium* and *Haemoproteus* parasites were isolated from three different mosquito species with clear differences in parasite prevalence. *Culex pipiens* showed, by far, the highest parasite prevalence, suggesting that this species probably play a central role in the transmission of blood parasites in the studied area. Molecular isolation of parasites from mosquitoes could be used to identify the occurrence of encounters between parasites and mosquitoes, although this not necessarily implies that insects are real vectors of the parasite lineages isolated, because the parasites can be unable to replicate in the salivary glands, that may be the case of *Haemoproteus* parasites [23]. Although there is no previous information on the role of wild mosquitoes in the transmission of avian malaria parasites in Italy, recent studies have isolated *Plasmodium* parasites from *Cx. pipiens* mosquitoes captured in Czech Republic [16], Portugal [18], Switzerland [15,17], and Spain [14]. Also, sporozoites of both *Plasmodium relictum* and *Plasmodium vaughani*, parasite lineages found in this study, have been previously isolated from *Cx. pipiens* mosquitoes [9,24]. Furthermore, the *Plasmodium* lineage LINN1 was isolated from whole un-engorged *Cx. pipiens* mosquitoes [25]. Curiously, we isolated avian blood parasites from two mosquito abdomens containing a non-avian derived blood meal. This could be due to the occurrence of undetected mixed blood meals in the mosquito, the presence of avian parasites in the blood on these vertebrate hosts (see [12]) or simply the fact that parasites isolated were in the mosquito tissue but not in the blood meal [26]. This last possibility is supported by the detection of parasite DNA in mosquitoes with no host identification due to degraded blood, and consequently where it is unlikely that the parasites detected come from the vertebrate blood.

The invasive mosquito *Ae. albopictus* preferably bites on mammals, especially on humans [27-29], which is clearly supported by results from this study. However, this species is able to feed on non-mammal species including birds, with birds compromising between 0.8 to 73.0% of the total blood meals identified in previous studies [30], potentially playing a role on the transmission of avian malaria parasites. Under laboratory conditions, development of avian *Plasmodium* sporozoites occurs in *Ae. albopictus* [31], see also [9]. Furthermore, avian malaria parasites have been isolated from wild *Ae. albopictus* mosquitoes, but usually showing low prevalence of infection [32-34]. Also, some studies have reported the absence of *Plasmodium* in *Ae. albopictus* mosquitoes from Japan [27,35]. Overall, results from these studies together with those reported here support that *Ae. albopictus* could be involved in the transmission of avian malaria parasites, although the risk of parasite spread by this mosquito species in Europe would be minimal due to its low biting rate on birds.

The use of molecular tools on avian malaria studies has allowed the identification of a broad diversity of parasite lineages infecting birds in different areas and, as a result, it is possible to infer their current geographical distribution. Most of the parasite lineages isolated in this study are widespread, being isolated from birds from different countries of the old world (Table 3, see Malavi database [36]). However, although the same parasite cyt b lineage could be found in birds from both Europe and Africa, as in the case of the widespread *Plasmodium* SGS1, the characterization of highly variable genes (i.e. merozoite surface protein 1 gene, MSP1) provided strong evidence of parasite differentiation among continents, and consequently parasites with the same sequence of cytb may in fact correspond to different lineages with more reduced distribution areas affecting the inference of geographical areas of parasite transmission [37].

In conclusion, our results support that, the risk of spread of avian malaria parasites by the invasive mosquito *Ae. albopictus* in Europe would be minimal. However, its ability to transmit other pathogens of sanitary importance including viruses and nematodes support the necessity of setting up an active surveillance and control programme on this species [1]. Further studies are necessary in order to identify those factors affecting infections by different avian pathogens (i.e., WNV and avian malaria parasites) in vertebrate [38] and invertebrate [39] hosts, which may determine parasite epidemiology.
